# The Non-Linear Profile of Aging: U-Shaped Expression of Myostatin, Follistatin and Intermediate Signals in a Longitudinal In Vitro Murine Cell Sarcopenia Model

**DOI:** 10.3390/proteomes12040034

**Published:** 2024-11-22

**Authors:** Janire Alonso-Puyo, Oihane Izagirre-Fernandez, Olatz Crende, Jesús Seco-Calvo, Ainhoa Fernandez-Atutxa, Diego Fernandez-Lazaro, Patricia Garcia-Gallastegi, Begoña Sanz

**Affiliations:** 1 Physiology Department, Faculty of Medicine and Nursery, University of the Basque Country (UPV/EHU), Barrio Sarriena, sn., 48940 Leioa, Spain; jalonso094@ikasle.ehu.eus (J.A.-P.); jesus.seco@unileon.es (J.S.-C.); patricia.garcia@ehu.eus (P.G.-G.); 2 Cell Biology and Histology Department, Basque Country University School of Medicine, Nursery University of the Basque Country (UPV/EHU), Barrio Sarriena, sn., 48940 Leioa, Spain; oizagirre002@ikasle.ehu.eus (O.I.-F.); olatz.crende@ehu.eus (O.C.); 3 Institute of Biomedicine (IBIOMED), Universidad de León, Vegazana Universitary Campus, 27071 León, Spain; 4 Nursery I Department, Basque Country University School of Medicine and Nursery, University of the Basque Country (UPV/EHU), Barrio Sarriena, sn., 48940 Leioa, Spain; ainhoa.fernandez@ehu.eus; 5 Biocruces Bizkaia Health Research Institute, 48903 Barakaldo, Spain; 6 Department of Cellular Biology, Genetics, Histology and Pharmacology, Faculty of Health Sciences, University of Valladolid, Campus of Soria, 42004 Soria, Spain; diego.fernandez.lazaro@uva.es; 7 Neurobiology Research Group, Faculty of Medicine, University of Valladolid, 47005 Valladolid, Spain

**Keywords:** sarcopenia, muscle, myoblasts, aging, myostatin, follistatin, mTOR, RPS6KB1, biomarkers, C2C12

## Abstract

Sarcopenia is linked to the decline in muscle mass, strength and function during aging. It affects the quality and life expectancy and can lead to dependence. The biological process underlying sarcopenia is unclear, but the proteins myostatin and follistatin are involved in the balance between muscle breakdown and synthesis. While myostatin promotes muscle breakdown, follistatin promotes muscle growth, but several works have shown an inconsistent association of these proteins with aging-related parameters in serum of older people. We aimed to know the evolution of these putative sarcopenia biomarkers along muscle aging in an in vitro model. We created and phenotyped a longitudinal murine model (C2C12 cells). Then, we analyzed the protein and genetic expression of myostatin and follistatin as well as the signaling pathway regulators mTOR and RPS6KB1. Myostatin and RPS6KB1 showed a similar tendency in both protein and genetic expression with aging (basal–up–down). Follistatin, on the other hand, shows the opposite tendency (basal–down–up). Regarding mTOR, the tendencies differ when analyzing proteins (basal–up–down) or genes (basal–down–down). Our work demonstrates a U-shape tendency for myostatin and follistatin and for the signaling pathway regulators. These results could be of the utmost importance when designing further research on seeking molecular biomarkers and/or targets for sarcopenia.

## 1. Introduction

Skeletal muscle is essential for human living given its function for the maintenance of posture, locomotion and breathing, as well as for its role as nutrient store and metabolic regulator [1,2]. Considering this, it is not surprising that the alteration in muscle mass and strength has an important role in some diseases and syndromes, playing a significant part in aging. Decreasing muscle mass, strength and function during aging interferes with the quality and expectancy of life [3]. In this way, sarcopenia has been defined as a progressive and generalized skeletal muscle disorder, characterized by a loss of muscle mass, strength and power [3,4].

Even if the biological process underlying sarcopenia is not very clear [5], it is known that myostatin and follistatin are implicated in the balance between the synthesis and degradation of skeletal muscle [6]. On the one hand, myostatin (also known as GDF8) is a secreted protein, expressed in fat, bone and muscle [7]. This myokine belongs to the transforming growth factor β (TGF-β) family and negatively regulates myogenesis [8,9,10]. On the other hand, follistatin is another secreted protein, broadly expressed and with a significant role during embryogenesis [11]. This protein is a myostatin-binding protein that can inhibit myostatin activity in vitro and promotes muscle growth in vivo [9,12]. Since myostatin and follistatin bind to the same receptor, the balance between them is associated with muscle atrophy or hypertrophy [13].

As myostatin promotes muscle degradation, we can expect higher myostatin levels in serum from older people, and to be serum levels inversely associated with muscle mass, as described in some works [14]. However, in other works [15,16], including ours in older people living in nursing homes [17,18] and in older people that had been hospitalized [19], this association is the opposite than the expected one. In the same vein, despite some works showing lower follistatin levels in older populations [20], some works [21] as well as ours [19] have also found the association to be opposite to expected. These apparently paradoxical results could be justified by subjacent counter regulatory mechanisms according to the theory that each organ produces a “chalone”, a factor limiting its own growth [22]. Chalones are tissue-specific substances that operate through a cell membrane receptor mechanism via a type of inhibitory paracrine control [23].

The mammalian target of rapamycin (mTOR) is a well-conserved serine/threonine kinase with a very important role in protein synthesis and degradation [24,25,26] that modulates muscle hypertrophy and muscle wastage [26]. Myostatin inhibits the mTOR pathway [27,28,29], whereas follistatin activates it [30]. Downstream of the mTOR signaling pathways is Rps6kb1 or p70S6K1 (p70S6), a protein kinase that promotes cell proliferation, growth and cell cycle progression [31,32]. In this way, the components of the Akt/mTOR/p70S6K1 pathway are lower in older people [33,34,35,36]. However, to our knowledge, there is no publication where both of these proteins (mTOR and Rps6kb1) are studied in relation to sarcopenia.

Given that the concentrations of myostatin and follistatin in the serum of older people is associated with aging-related parameters in the opposite way than expected, we wondered if this association follows a linear pattern across aging process, or if it follows a U-shaped pattern. Unfortunately, carrying out such longitudinal research in human beings presents time, economic and methodologic limitations [37,38]. Moreover, results related to serum proteome analysis do not give us information related to the regulatory mechanisms inside the cell. Recently, we published a narrative review about experimental tools for research on sarcopenia [39]. Thus, primary myoblast cultures, such as C2C12, would be the most interesting cells to study sarcopenia [40].

In this work, we aimed to generate and phenotype a three-phase in vitro longitudinal sarcopenia model with C2C12 murine cells. We also aimed to look into the proteome by Western blotting and to compare the genetic expression of myostatin, follistatin, mTOR and RPS6KB1 alongside the in vitro aging of myoblasts. In this way, we could gain information about the evolution of these putative molecular biomarkers of sarcopenia alongside muscle aging process as well as increase the knowledge on muscle related signaled molecules.

## 2. Materials and Methods

### 2.1. Sarcopenia Model Generation

This study was approved by The Biological Agents Committee of the University of the Basque Country (CEIAB: M30-2021-239, M30-2024-118).

C2C12 murine skeletal myoblast cells were used to generate the sarcopenia model in vitro following the protocol published by Sharples et al. (2011) [41]. Cells were purchased from ThermoFisher and grown in DMEM with 10% FBS, 10% NCS, 1% L-glutamine (2 mM final), and 1% penicillin–streptomycin solution, maintaining the cells’ undifferentiated state. The cells were seeded in T75 until ~70% of confluence; when the confluence was achieved, passages were deemed complete. During the cell culture process, cells were collected, establishing three groups for analysis. Cells from the second passage (P2) were considered no-sarcopenic/control cells (C), and cells from the intermediate and last passages (P13 and P23) were classified as sarcopenia (S) and severe sarcopenia (SS) cells, respectively.

The sarcopenia longitudinal model was characterized by aged passages of C2C12 cells from the purchased vial to P23 along 56 days. Cells underwent the following timeline for the phases of analysis: control cells were collected seven days after the initial seeding of the purchased vial, sarcopenia cells on day 33, and the severe sarcopenia cells on day 56.

### 2.2. Cell Staining

After the cells were collected in the established groups, they were seeded in round slides and incubated for 24 h until adherence was achieved. Adhered cells were fixed with 4% paraformaldehyde (PFA) and maintained in PBS until staining was performed.

Cells were stained following the standard hematoxylin–eosin protocol, and images were then captured using an inverted microscope (Zeiss microscope) with the 20× objective. Each group was captured 5 times, and the characteristics were determined individually in each group.

### 2.3. Protein Extraction and Western Blot (WB)

C2C12 cell protein extraction and Western blot were realized by following the protocol published by Uribe-Etxebarria et al., 2020 [42]. For protein detection, we used anti-Nup153 (1:1000, Novus Biologicals NBP1-81725), anti-Myostatin (Mstn, 1:200, Santa Cruz Biotechnology: sc-134345), anti-Follistatin (Fstl3, 1:100, sc-365003), anti-mTOR (1:200, sc-517464), and anti-Rps6kb (1:100, sc-377529).

The secondary antibodies used were anti-mouse -HRP 1:2000 in TBST-BSA 5% (Ab6729, Abcam, Cambridge, UK) and anti-rabbit-HRP 1:2000 in TBST-BSA 5% (BA1054, Boster Bio, Pleasanton, CA, USA), which were incubated for 90 min. The blots were developed using Luminata Crescendo Western HRP Substrate (WBLUR0500 Millipore, Burlington, MA, USA). Western blot images were taken in a Syngene G: BOX CHEMI XR5system (Syngene, Cambridge, UK). The membranes were stripped using Red Blot (M2504, Inmmobilon^®^ EMD Millipore, Burlington, MA, USA). Samples were quantified by Fiji-ImageJ after background subtraction.

### 2.4. RNA Extraction, Reverse Transcription, and Quantitative Real-Time PCR (qPCR)

RNA from the cells was extracted with the RNeasy Plus mini-Kit (QIAGEN) commercial Kit, following the steps indicated by the manufacturer, and the quantification and purity were analyzed by spectrophotometry with NanoDrop (Synergy HT, BioTek^®^, Agilent, Santa Clara, CA, USA). cDNA was obtained by retrotranscription, following the recommendations of the commercial iScriptTM cDNA Synthesis Kit (BIO-RAD, Hercules, CA, USA) in a thermal cycler (MyCycler, BIO-RAD).

mRNA expression was detected in a CFX96 Real-Time System Thermal Cycler (BIO-RAD) using qPCR SYBR^®^ Green Master Mix (Thermo Fisher Scientific, Waltham, MA, USA). Primers were purchased in KiCqStart^®^ (Sigma-Aldrich, Burlington, MA, USA); the forward and reverse sequences are shown in Table 1. One housekeeping gene, *gapdh*, was used to normalize the gene expression.

Quantification of the data obtained from the thermocycler, namely Ct values, was carried out by normalizing them to the house-keeping gene, calculated as ΔCt. ΔCt values were then transformed to relative fold changes in gene expression between the experimental conditions [43]. The differences between the compared conditions were considered statistically significant at a confidence interval of 95%. The experiment was repeated at least three times, and the graphs show the means ± SEM.

### 2.5. Statistical Analysis

Statistical analysis was performed using the IBM SPSS Statistics 29 software package (SPSS, Inc., Chicago, IL, USA). All experiments were performed in triplicate. The mean and standard deviation were calculated, and to compare the genic expression, P2/C values were assigned to one, and P13/S and P23/SS values relativized to P2/C. The relativized means were compared (P2/C vs. P13/S; P2/C vs. P23/SS; P13/S vs. P23/SS) using Student’s *t*-tests for repeated measures. The difference between groups was considered significant at *p* < 0.05.

## 3. Results

### 3.1. Phenotype Characterization of C2C12 Cells Along Passages

**Figure 1:**
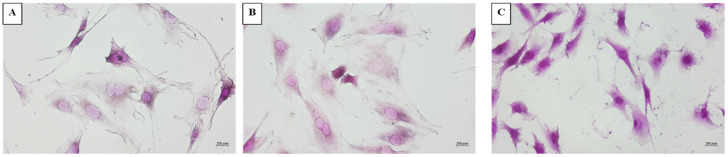
C2C12 murine cell sarcopenia model representative images stained with hematoxylin–eosin. (**A**): Control (P2/C). (**B**): Sarcopenia (P13/S). (**C**): Severe sarcopenia (P23/SS).

Figure 1 shows the images of the C2C12 sarcopenia model study groups (P2/C, P13/S, P23/SS). Regarding cellular shape, in both control group (P2/C, Figure 1A) and the sarcopenia group (P13/S, Figure 1B), the observed cells showed longer cellular extensions in comparison to the severe sarcopenia group (P23/SS, Figure 1C). In addition, in P2/C and P13/S cells (Figure 1A,B), the cells appear in division with two nuclei in them, whereas in the severe sarcopenia group (Figure 1C), the observed cells show only one nucleus.

In terms of the staining, hematoxylin is a basic dye that binds to acidic substances in the cell, such as ribosomes or the genetic material found within the nucleus, dying them deep blue/violet. In the first passages (P2/C, Figure 1A), the cells presented nuclei that are uniform in size and stained with hematoxylin, showing fairly well-organized chromatin. As passages increased and cells aged, the nuclei can become larger, as seen in Figure 1B (P13/S), showing a denser and more aggregated chromatin, characteristics of senescence. An increment in the number of nucleoli can also be observed, indicative of cellular stress and greater transcriptional activity (P23/SS, Figure 1C).

Regarding the cytoplasm, differences also appeared among the three steps. In this case, we used eosin to stain the basic substances of the cell, such as the cytoplasm. In P2/C (Figure 1A), the cytoplasm is only stained with eosin, achieving a pinker color. However, as the cells aged, the cytoplasm not only presents a darker color, as shown in P23/SS (Figure 1C), in which it becomes more purple in color, but also appears more granular. Aged cells can accumulate more acidic substances in the cytoplasm, such as RNA. One of the reasons that this may occur is due to an increase in protein synthesis in response to stress or an accumulation of acid residues due to a decrease in cellular degradation, both processes associated with aging. In turn, senescent cells usually show an increase in transcriptional activity and the production of both rRNA and mRNA, which leads to an increase in the amount of acidic material in the cytoplasm, thus staining with hematoxylin.

### 3.2. Antagonistic Expression of Myostatin and Follistatin

**Figure 2:**
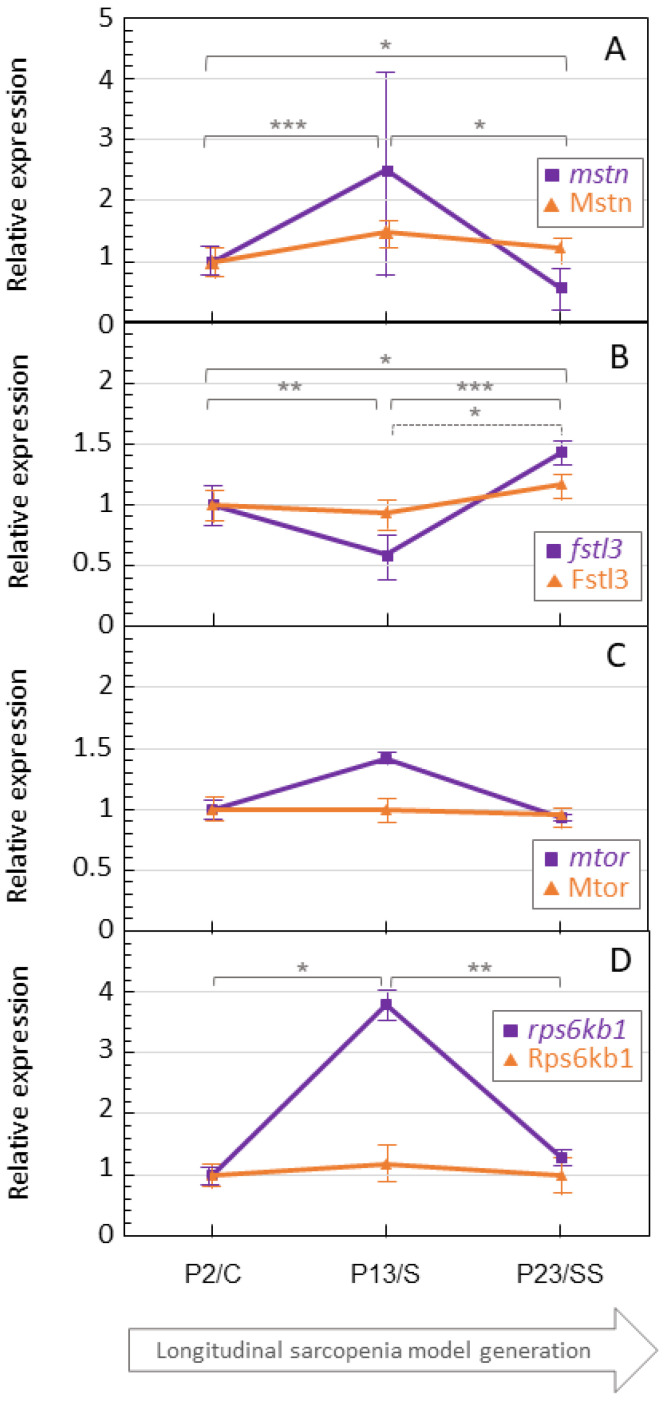
Relative gene (□) and protein (Δ) expression of myostatin (**A**), follistatin (**B**), mTOR (**C**), and RPS6KB1 (**D**) along the C2C12 sarcopenia model. Mean and standard deviation are shown for each point. * *p* < 0.05, ** *p* < 0.01, *** *p* < 0.001. Significance between data pairs is shown with solid lines for gene expression and dotted lines for protein expression.

Protein and gene expressions of myostatin and follistatin were analyzed along the sarcopenia model. Raw data on gene and protein expressions are shown in the Supplementary Data (Supplementary File S1) and Supplementary Figure S1. For both, P2/C obtained values assumed as one, and P13/S, P23/SS values were relativized to P2/C. For protein expression, the WB results were also normalized to Nup in order to minimize changes in protein loading in each gel. Figure 2A shows results for myostatin, and Figure 2B for follistatin.

Myostatin protein expression was increased from P2/C cells to P13/s cells, and it decreased at the final stage (P23/SS). Thus, myostatin expression alongside cell aging follows an inverted U-shaped profile (basal–up–down). On the contrary, the expression of follistatin follows a U-shaped profile (basal–down–up). Despite the clear differences in the visual data, differences were only significant in the case of follistatin P13/S vs. P23/SS.

The gene expressions of myostatin and follistatin along the sarcopenia model, as in the analysis of protein expression, showed an opposite profile for both molecules: an inverted U-shape for myostatin (basal–up–down) and a U-shape for follistatin (basal–down–up). In this case, variations were significant for both genes between all the comparations.

### 3.3. Alteration in Major Molecules of the Muscular Proliferation and Degradation Signaling Pathway

mTOR (Figure 2C) and RPS6KB1 (Figure 2D) relative protein and gene expressions were also analyzed due to the implication of both molecules in muscular proliferation and degradation signaling pathways, where myostatin and follistatin take part. The protein expression shown in both cases (for Mtor and Rps6kb1) followed an inverted U-shape tendency (basal–up–down); however, there were not significant differences between groups. The corroboration of the results at a genetic level showed that the relative *mtor* expression decreased between the groups (basal–down–down). However, the differences were not significant. Regarding *rps6kb1* gene expression, it resembles the results obtained in the protein expression analysis, and it looks similar to myostatin’s expression tendency. There was an increase between P2 and P13 and a decrease between P13 and P23. The increase between P2 and P13, and the decrease between P13 and P23, have significant *p* values.

## 4. Discussion

In this work, we developed an in vitro sarcopenia model using murine C2C12 cells, slightly modifying a pre-existing published protocol. We analyzed cellular morphological changes alongside the aging process, as well as the protein and gene expression of molecules related to sarcopenia, such as myostatin and follistatin. We also analyzed mTOR and RPS6KB1, intermediate signaling molecules. Our results revealed significant differences not previously described in the protein and genetic expression of these molecules along the three studied cellular passes (P2/C, P13/S, and P23/SS).

The phenotypic study reveals a senescence-associated secretory phenotype (SASP) during the C2C12 sarcopenia-like studied groups (control, P2/C; sarcopenia, P13/S; and severe sarcopenia, P23/SS). The control group and mild sarcopenia group showed larger cellular extensions in comparison to the severe sarcopenia group. This reduction in the cellular extensions might result from the senescence of the cells [44]. Moreover, during the cell aging process, the nuclei of the cells have a different appearance. Alongside the passages, the nuclei become larger and show denser chromatin, indicative of the senescence process they are undergoing. During the aging process, the cells have been described to firstly enlarge in size [45,46] and then reduce in size in order to undergo programmed cell death [47,48]. The C2C12 cell aging process is related to a reduction in the proliferation rate. This reduction in the proliferation rate has already been described in other types of cells, such as fibroblast [49,50]. Age-related oxidative stress increment is known to be caused by the reduction in the availability of antioxidants, which leads to a deterioration of the cells [51,52]. Moreover, other authors have described that mesenchymal stem cells show that oxidative stress induce cell senescence and deterioration, even causing an accumulation of granular cytoplasmic inclusions [53]. Similar results have been obtained in our cell model, as the severe sarcopenia group shows an increment in the number of nuclei as well as a higher hematoxylin staining due to the granular cytoplasm, which might appear due to the higher level of the oxidative stress, related to a higher protein synthesis and accumulation of acidic residues [54].

Our results with in vitro cultures demonstrate that the muscle synthesis-related molecules myostatin and follistatin show an inverted-U-shape profile and a U-shape profile, respectively. Regarding myostatin, both when studying protein and gene expression, intermediately aged cells (P13/S) showed higher expression levels. These results agree with the proposed role of myostatin as an inhibitor of muscle synthesis. However, as we hypothesized from others and our previous results obtained from older people’s serum, myostatin levels (at the protein and gene expression levels) were lower at the eldest stage of our model (P23/SS). Therefore, we can propose that along the aging process (at least in our in vitro model), myostatin does not follow a linear or J-shape pattern but a U-shaped one (basal–up–down). This is probably the consequence of counter-regulatory mechanisms and/or its putative action as a chalone. In the case of follistatin, the obtained pattern was just the opposite: firstly, it is low, then it increases, and then it reduces again with severe sarcopenia (basal–up–down).

Both of these molecules have a limited research on their analysis in cell culture. Both primary myoblasts and cell lines have been used to study the effect of the addition of recombinant myostatin in cell cultures to inhibit proliferation [55]. On the other hand, analyses on mice models have shown that myostatin overexpression led to cachexia, a wasting syndrome associated with muscle wasting [56,57]. In addition, Sriram and his colleges (2011) [55] have demonstrated that Mstn-/- C57Bl/6J mice muscle mass is higher compared to the wild C57Bl/6J mice. Winbanks and her colleagues (2012) [30] took into account the relation between follistatin and myostatin and analyzed the effect of the overexpression of follistatin in myostatin-null mice. Their study shows that overexpression of follistatin led to the hypertrophy of the skeletal muscle via the mTOR/p70S6K pathway.

These biomolecules present some proteome complexity. On the one hand, myostatin proteoforms form a latent complex containing mature protein and propeptides [9,58,59]. On the other hand, follistatin presents a high proteome complexity due to the similarities in the follistatin-like proteins, for instance, activin A-follistatin muscle activation complex only shows a stable complex with Fstl3 [60]. In addition, both mTOR and Rps6kb1 protein species have an extended proteome complexity, which leads to the unique multifaceted functions both proteins carry out [61,62].

Physical frailty is an age-related syndrome characterized by a progressive decline in physiological systems; sarcopenia has been proposed as one of the major components of frailty [63]. Due to its implications in the human quality of life, most of the studies about sarcopenia and frailty with myostatin or follistatin have been developed in human serum/plasma. Some studies found a higher concentration of myostatin in both older men and women with frailty [14], while other studies have observed a higher serum myostatin level in non-frail individuals [17]. The difference between these studies is due to the heterogeneity of the sample. Arrieta et al. (2018) [64] worked with an older and frailer population compared to the other studies, and considering that myostatin is secreted by skeletal muscle, participants with lower muscle mass may express less myostatin. Another study found that while the concentration of myostatin was lower in people with frailty or sarcopenia, the level of follistatin was higher in people with frailty [19], so a negative association was observed between myostatin and frailty, and sarcopenia. Follistatin, on the other hand, was positively associated with frailty [19] and sarcopenia [65]. Studies realized on aged human serums and our results on C2C12 murine cells show a similar tendency, where myostatin is downregulated and follistatin is upregulated in severe sarcopenia. In order to be able to use a molecule as a biomarker of aging, it is very important to know exactly what pattern these molecules follow over time. The fact that aging and the associated molecular changes (multi-omics) do not follow a linear pattern, a wave pattern, has been reported by different authors [66,67]. This fact represents a milestone in the search for biomarkers and therapeutic targets. Using myostatin and/or follistatin as sarcopenia biomarkers requires further knowledge of the way they evolve alongside muscle aging.

Major molecules of the muscular proliferation and degradation signaling pathway, such as mTOR and Rps6kb1, showed different profiles in our study. On the one hand, mTOR shows a slight decrease between every group, showing a J-shape profile. On the other hand, Rps6kb1 shows an inverted U-shape profile. Picca et al. (2021) [68] have described a U-shape association between mTOR and cognitive function throughout the aging process. However, in this work, protein expression was not consistent with the U-shape profile theory, as mTOR protein levels decreased with cell aging. In the case of the *rps6kb1* gene, we expected similar results to those obtained in the previous case (mTOR), as this gene encodes a member of the serine/threonine kinase family of ribosomal kinase S6 (p70s6k1), an effector protein of mTOR, since the activation of mTOR leads to the activation of p70s6k [69]. Thus, we did not achieve the expected results, as mTOR protein expression shows a J-shape profile and Rps6kb1 a U-shape profile. This is why mTOR and Rps6kb1 can be associated with severe sarcopenia positively and negatively, respectively. Nevertheless, the knowledge in relation to these two molecules is insufficient as we have not found further studies of these two molecules among sarcopenia or other muscle disorders, through neither cell culture, animal models, or human serum.

The muscle proliferation process is regulated by the Akt/mTOR/RPS6K pathway. Both myostatin and follistatin bind to one of the Akt/mTOR signaling pathway receptors, activating or inhibiting muscle cell proliferation. Follistatin is known to be an inhibitor of myostatin [9,12]. That is way the binding of myostatin to the receptor inhibits the activation of the Akt/mTOR/RPS6K pathway, inhibiting myoblast differentiation and myotube size [70,71,72]. Moreover, other authors have demonstrated that the binding of follistatin to the same myostatin receptor activates Akt/mTOR/p70S6K1/S6 signaling, enhancing muscle growth [26,30]. Our results measuring gene and protein expression in the sarcopenia model reveal opposite expression patterns in myostatin and follistatin, an inverted U-shape and a U-shape, respectively, which may be associated with their different functional activity towards muscle degradation and activity in the Akt/mTOR/p70S6K1/S6 pathway.

To our knowledge, the study presented here is the first to analyze this combination of molecules in C2C12 cells in the sarcopenia research area. By combining molecules that have functions linked to muscle in one way or another and that are all affected by aging, we can gain a new perspective on what happens in cells during this process. Thus, not only can we understand the function of each one of them individually, but we can also analyze the relationship between them in order to better understand both the metabolic pathways and the markers themselves. In addition, using murine myoblasts that are quite similar to human muscle cells gives us the opportunity to carry out this project in the long term in a controlled manner and without the need for biopsies, which saves us encountering ethical and methodological issues. Other sarcopenia cell models described by Mankhong and her colleges (2020) [40] are based on the supplementation of different molecules such as H_2_O_2_ or ceramide, among others, to induce the aging process in cells, which could alter the molecular mechanisms of the cells. However, our model, which is based on the work conducted by Sharples et al. (2011) [41], allows us to investigate a longitudinal model without adding chemical factors, which better represents the in vivo cell environment. Thus, this project identifies possible sarcopenia biomarkers in order to facilitate the diagnosis and treatment of this pathology.

Despite the above-mentioned strengths, the work also presents several limitations. Our results confirm the hypothesis based on the results obtained in older people. However, the main limitation of our results is that they were obtained in an in vitro model from murine cells. It is possible that our conclusions can be transferred to more complex real systems, but in this sense, they should be taken with caution when making proposals in clinical practice. On the other hand, this limitation could be diminished by setting up more complex systems such as 3D cellular models, organoids, animal models, or computational models that help us to confirm and refine our hypothesis. In addition, due to the proteoforms and preoteome complexity of the molecules analyzed, further research needs to be performed in order to clarify the changes in these biomolecules with aging.

Finally, taking into account the obtained results, we can conclude that developing an experimental project based on the C2C12 murine myoblasts could be a complementary tool in order to study the molecular aspects of the development of sarcopenia. This could also help to detect possible useful biomarkers for the development of early diagnosis, monitoring, and treatments tools for this pathology. Moreover, the results obtained in this analysis of molecular expression support the hypothesis that during aging, there is an up-regulation of the compensatory system of the organism. The compensatory system may increase or decrease the regulation of biomolecules and can be represented with different graphic profiles, such as the previously defined U-shape, inverted U-shape, and J-shape profiles.

## 5. Conclusions

Myostatin, follistatin, and intermediate signals as mTOR and Rps6kb1 evolve with aging, but not following a linear pattern. In the case of myostatin, follistatin, and Rps6kb1, proteomic expression and gene expression fit the obtained pattern (basal–up–down, basal–down–up, and basal–up–down, respectively). For mTOR, despite having a basal–up–down pattern of protein expression, its gene expression does not fit. Given the wide role of mTOR in aging-related processes, further studies should be developed in order to uncover the subjacent regulatory mechanism of all of these changes. On the whole, these results are of the utmost importance, since they confirm the non-linear evolution of several molecules slightly linked to sarcopenia with muscle aging. Research on this area presents two branches of attention: on the one hand, to further deepen the regulatory mechanisms using experimental in vitro models; on the other hand, to validate these results with results obtained in in vivo models, including animal models and in human beings. Our work confirms the gap between clinical and basic research. Finally, we believe that our results could serve to explain some of the controversies that currently exist in the literature, for example, some of those that exist on the association between serum myostatin and follistatin concentration in older people and parameters associated with deterioration in physical function and loss of muscle function.

## Figures and Tables

**Table 1:** Primer pairs sequence (F: forward, R: reverse) of the transcripts of interest and the housekeeping, annealing temperature (°C) and amplicon size (bp).

Primer	Sequence (5′-3′)	Annealing Temperature		Amplicon Size
Myostatin (*mstn*)	F: CTATAAGACAACTTCTGCCAAG	57.3	52.4	157
	R: AGAAAGTCAGACTCTGTAGG			
Follistatin (*fstl3*)	F: GTCAAAAGTCTTGCGCTC	58.7	56.3	160
	R: GAGATGTAGGTAACGTTGTTG			
Mechanistic target of rapamycin (*mtor*)	F: CTTCACAGATACCCAGTACC	56.0	54.0	137
	R: AGTAGACCTTAAACTCCGAC			
Ribosomal Protein S6 Kinase B1 (*rps6kb1*)	F: AAAGGGATCATCTACAGAGAC	56.3	60.2	153
	R: AGGGGCCATGTATTCTATTG			
Glyceraldehyde 3-phosphate dehydrogenase (*gapdh*)	F: CCAGTATGACTCCACTCACG	57.43	57.80	153
	R: GACTCCACGACATACTCAGC			

## Data Availability

Data supporting reported results can be found at Supplementary Materials.

## Supplementary Materials

The following supporting information can be downloaded at: https://www.mdpi.com/article/10.3390/proteomes12040034/s1, Figure S1: Representative images of NUP (Nup), Myostatin (Mstn), Follistatin (Fstl3), mTOR (Mtor) and RPS6KB1 (Rps6kb1) protein expression by Western blot for the longitudinal C2C12 sarcopenia model; File 1: Raw data; File 2: original images of Western Blot.
