# Supplementary material for: The Non-Linear Profile of Aging: U-Shaped Expression of Myostatin, Follistatin and Intermediate Signals in a Longitudinal In Vitro Murine Cell Sarcopenia Model
Source: Proteomes. 2024 Nov 22;12(4):34. doi: 10.3390/proteomes12040034 (PMC11587466; doi:10.3390/proteomes12040034)
Supplement: Supplementary file 1 [file proteomes-12-00034-s001.zip › Alonso-Puyo PROTEOMES Figure S1.pdf]

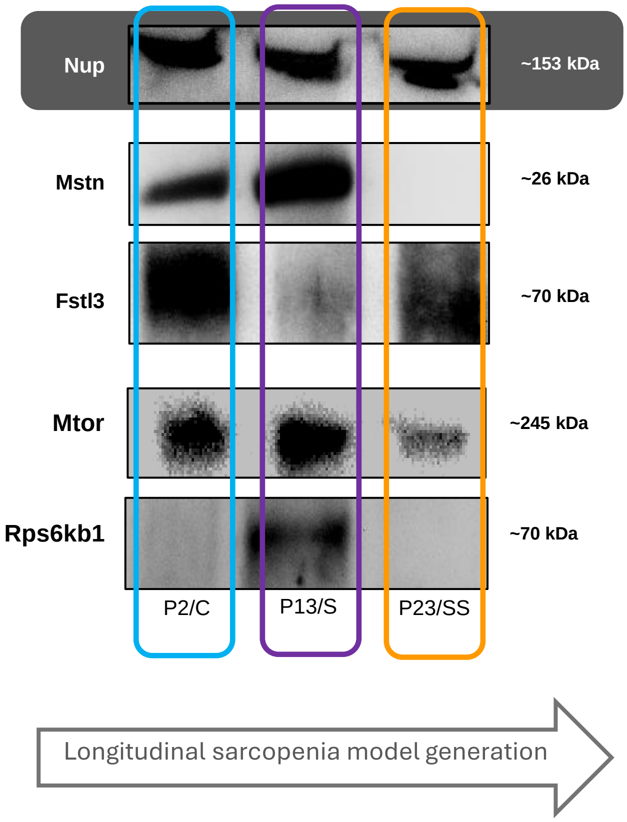

Supplementary Figure 1: Representative images of NUP (Nup), Myostatin (Mstn), Follistatin (Fstl3), mTOR (Mtor) and RPS6KB1 (Rps6kb1) protein expression by Western blot for the longitudinal C2C12 sarcopenia model.
