# Supplementary material for: The Non-Linear Profile of Aging: U-Shaped Expression of Myostatin, Follistatin and Intermediate Signals in a Longitudinal In Vitro Murine Cell Sarcopenia Model
Source: Proteomes. 2024 Nov 22;12(4):34. doi: 10.3390/proteomes12040034 (PMC11587466; doi:10.3390/proteomes12040034)
Supplement: Supplementary file 1 [file proteomes-12-00034-s001.zip › Alonso-Puyo PROTEOMES File 1 RAW DATA.pdf]

Well	Fluor	Target	Content	Sample	Cq
A01	SYBR	GAPDH	Unkn	p2	21.59
A02	SYBR	GAPDH	Unkn	p2	21.58
A03	SYBR	GAPDH	Unkn	p2	21.14
A04	SYBR	GAPDH	Unkn	p13	20.36
A05	SYBR	GAPDH	Unkn	p13	20.51
A06	SYBR	GAPDH	Unkn	p13	19.64
A07	SYBR	GAPDH	Unkn	p23	22.55
A08	SYBR	GAPDH	Unkn	p23	22.75
A09	SYBR	GAPDH	Unkn	p23	22.37
A10	SYBR	GAPDH	Unkn	H2O	39.01
A11	SYBR	GAPDH	Unkn	H2O	37.90
A12	SYBR	GAPDH	Unkn	H2O	38.28
C01	SYBR	RPS6KB1	Unkn	p2	24.69
C02	SYBR	RPS6KB1	Unkn	p2	24.73
C03	SYBR	RPS6KB1	Unkn	p2	24.20
C04	SYBR	RPS6KB1	Unkn	p13	24.15
C05	SYBR	RPS6KB1	Unkn	p13	23.95
C06	SYBR	RPS6KB1	Unkn	p13	23.39
C07	SYBR	RPS6KB1	Unkn	p23	25.57
C08	SYBR	RPS6KB1	Unkn	p23	25.66
C09	SYBR	RPS6KB1	Unkn	p23	25.56
C10	SYBR	RPS6KB1	Unkn	H2O	
C11	SYBR	RPS6KB1	Unkn	H2O	
C12	SYBR	RPS6KB1	Unkn	H2O	
D01	SYBR	FSTL3	Unkn	p2	26.74
D02	SYBR	FSTL3	Unkn	p2	26.47
D03	SYBR	FSTL3	Unkn	p2	26.25
D04	SYBR	FSTL3	Unkn	p13	25.10
D05	SYBR	FSTL3	Unkn	p13	24.99
D06	SYBR	FSTL3	Unkn	p13	24.48
D07	SYBR	FSTL3	Unkn	p23	28.52
D08	SYBR	FSTL3	Unkn	p23	28.46
D09	SYBR	FSTL3	Unkn	p23	28.31
D10	SYBR	FSTL3	Unkn	H2O	39.31
D11	SYBR	FSTL3	Unkn	H2O	39.57
D12	SYBR	FSTL3	Unkn	H2O	38.13
G01	SYBR	mTor	Unkn	p2	30.21
G02	SYBR	mTor	Unkn	p2	30.57
G03	SYBR	mTor	Unkn	p2	29.27
G04	SYBR	mTor	Unkn	p13	29.06
G05	SYBR	mTor	Unkn	p13	28.77
G06	SYBR	mTor	Unkn	p13	28.48
G07	SYBR	mTor	Unkn	p23	30.73
G08	SYBR	mTor	Unkn	p23	30.92
G09	SYBR	mTor	Unkn	p23	30.50
G10	SYBR	mTor	Unkn	H2O	41.03

G11	SYBR	mTor	Unkn	H2O	
G12	SYBR	mTor	Unkn	H2O	36.25
H01	SYBR	MSTN1	Unkn	p2	30.69
H02	SYBR	MSTN1	Unkn	p2	30.72
H03	SYBR	MSTN1	Unkn	p2	30.72
H04	SYBR	MSTN1	Unkn	p13	33.31
H05	SYBR	MSTN1	Unkn	p13	34.09
H06	SYBR	MSTN1	Unkn	p13	34.36
H07	SYBR	MSTN1	Unkn	p23	34.12
H08	SYBR	MSTN1	Unkn	p23	33.10
H09	SYBR	MSTN1	Unkn	p23	34.22
H10	SYBR	MSTN1	Unkn	H2O	42.16
H11	SYBR	MSTN1	Unkn	H2O	39.95
H12	SYBR	MSTN1	Unkn	H2O	42.28

Nup	Area	Mean	Min	Max	IntDen	RawIntDen
**P2**	1144	1875.791	1139	3225	2145905	2145905
**P13**	1144	1980.985	1374	2849	2266247	2266247
**P23**	1144	793.642	433	13458	907926	907926

Mstn	Area	Mean	Min	Max	IntDen	RawIntDen
**P2**	1248	1973.002	849	2779	2462306	2462306
**P13**	1248	2126.766	622	3108	2654204	2654204
**P23**	1248	1034.393	598	1579	1290922	1290922

Nup	Area	Mean	Min	Max	IntDen	RawIntDen
**P2**	5185	1021	320	2880	5294164	5294164
**P13**	5185	759	177	1917	3936327	3936327
**P23**	5185	775	165	2688	4020546	4020546

Mstn	Area	Mean	Min	Max	IntDen	RawIntDen
**P2**	4896	1768	162	5743	8660760	8660760
**P13**	4896	4459	486	11943	21835069	21835069
**P23**	4896	239	70	784	1171643	1171643

Nup	Area	Mean	Min	Max	IntDen	RawIntDen
P2	1144	1875.791	1139	3225	2145905	2145905
P13	1144	1980.985	1374	2849	2266247	2266247
P23	1144	793.642	433	13458	907926	907926

Fstl3	Area	Mean	Min	Max	IntDen	RawIntDen
P2	2464	3627.095	1448	4964	8937162	8937162
P13	2464	1755.63	800	2872	4325873	4325873
P23	2464	2966.915	1204	4875	7310478	7310478

Nup	Area	Mean	Min	Max	IntDen	RawIntDen
P2	5185	1021	320	2880	5294164	5294164
P13	5185	759	177	1917	3936327	3936327
P23	5185	775	165	2688	4020546	4020546

Fstl3	Area	Mean	Min	Max	IntDen	RawIntDen
P2	4402	2927	736	4964	12888102	12888102
P13	4402	1465	561	2872	6450988	6450988
P23	4402	2445	559	4875	10765503	10765503

Nup	Area	Mean	Min	Max	IntDen	RawIntDen
P2	3744	5174.341	665	9654	19372734	19372734
P13	3744	3127.595	430	7567	11709714	11709714
P23	3744	4790.806	173	4105	12960779	12960779

Mtor	Area	Mean	Min	Max	IntDen	RawIntDen
P2	3496	1151.47	731	1640	4025540	4025540
P13	3496	1510.035	840	2151	5279083	5279083
P23	3496	1542.019	1054	2493	5390898	5390898

Nup	Area	Mean	Min	Max	IntDen	RawIntDen
P2	5185	1021	320	2880	5294164	5294164
P13	5185	759	177	1917	3936327	3936327
P23	5185	775	165	2688	4020546	4020546

Mtor	Area	Mean	Min	Max	IntDen	RawIntDen
P2	980	644	257	1548	631991	631991
P13	980	554	208	1163	542994	542994
P23	980	368	165	635	361586	361586

Nup						
	Area	Mean	Min	Max	IntDen	RawIntDen
P2	3744	5174.341	665	9654	19372734	19372734
P13	3744	3127.595	430	7567	11709714	11709714
P23	3744	790.806	173	2105	2960779	2960779

Rps6kb1						
	Area	Mean	Min	Max	IntDen	RawIntDen
P2	1152	1014.765	437	2800	1169009	1169009
P13	1152	2613.177	1034	3924	3010380	3010380
P23	1152	446.055	124	9473	513855	513855

Nup						
	Area	Mean	Min	Max	IntDen	RawIntDen
P2	5185	1021	320	2880	5294164	5294164
P13	5185	759	177	1917	3936327	3936327
P23	5185	775	165	2688	4020546	4020546

Rps6kb1						
	Area	Mean	Min	Max	IntDen	RawIntDen
P2	6300	435	126	738	2740911	2740911
P13	6300	1152	229	2431	7260271	7260271
P23	6300	182	53	356	1150317	1150317
