# Supplementary figures and images for: The Non-Linear Profile of Aging: U-Shaped Expression of Myostatin, Follistatin and Intermediate Signals in a Longitudinal In Vitro Murine Cell Sarcopenia Model
Source: Proteomes. 2024 Nov 22;12(4):34. doi: 10.3390/proteomes12040034 (PMC11587466; doi:10.3390/proteomes12040034)

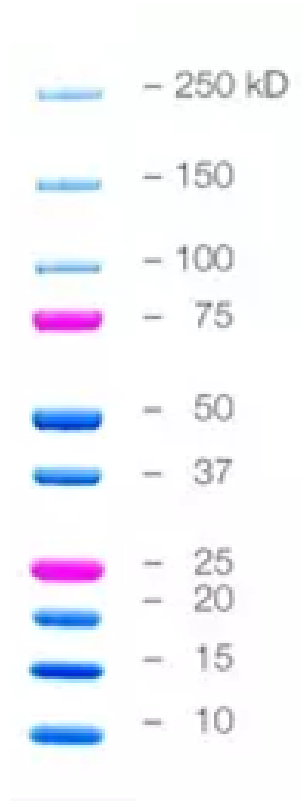

MW(kD)

150  
100  
75  
50  
37  
25  
20  
10

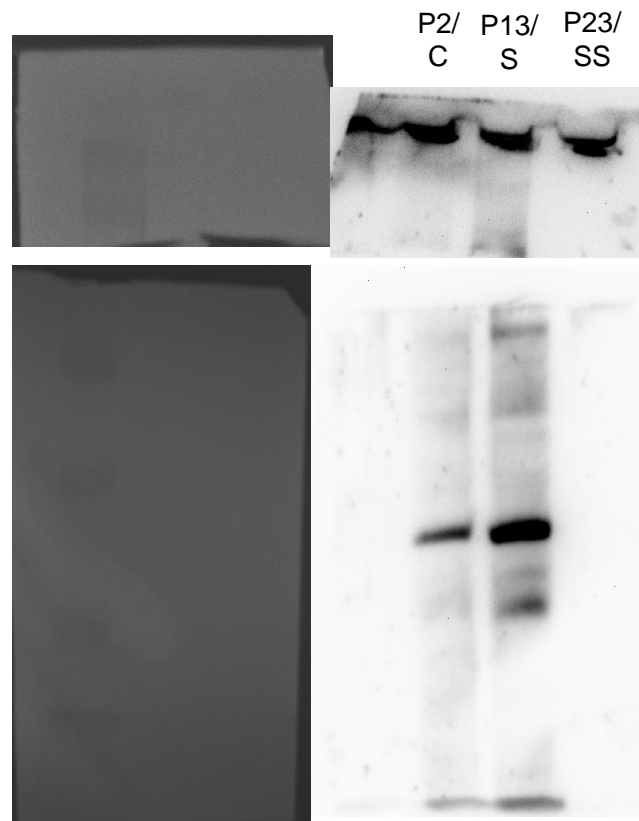

~153 kDa Nup

~26 kDa Mstn

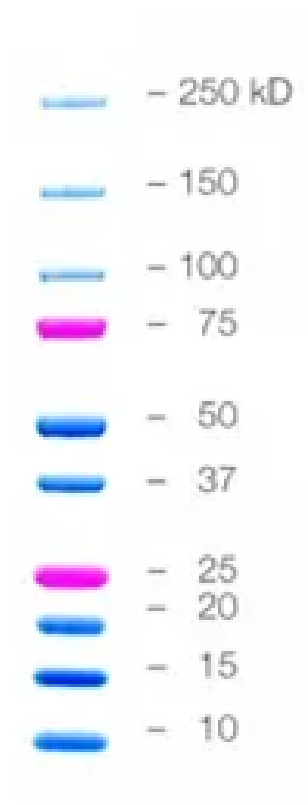

MW(kD)

150

100

75

50

37

25

P2/  
C

P13/  
S

P23/  
SS

~153 kDa

Nup

~70 kDa

Fstl3

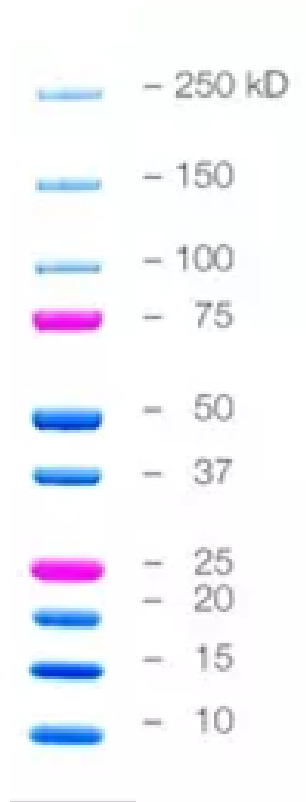

MW(kD)

250

150

100

75

50

37

25

P2/  
C

P13/  
S

P23/  
SS

~245 kDa

Mtor

~153 kDa

Nup

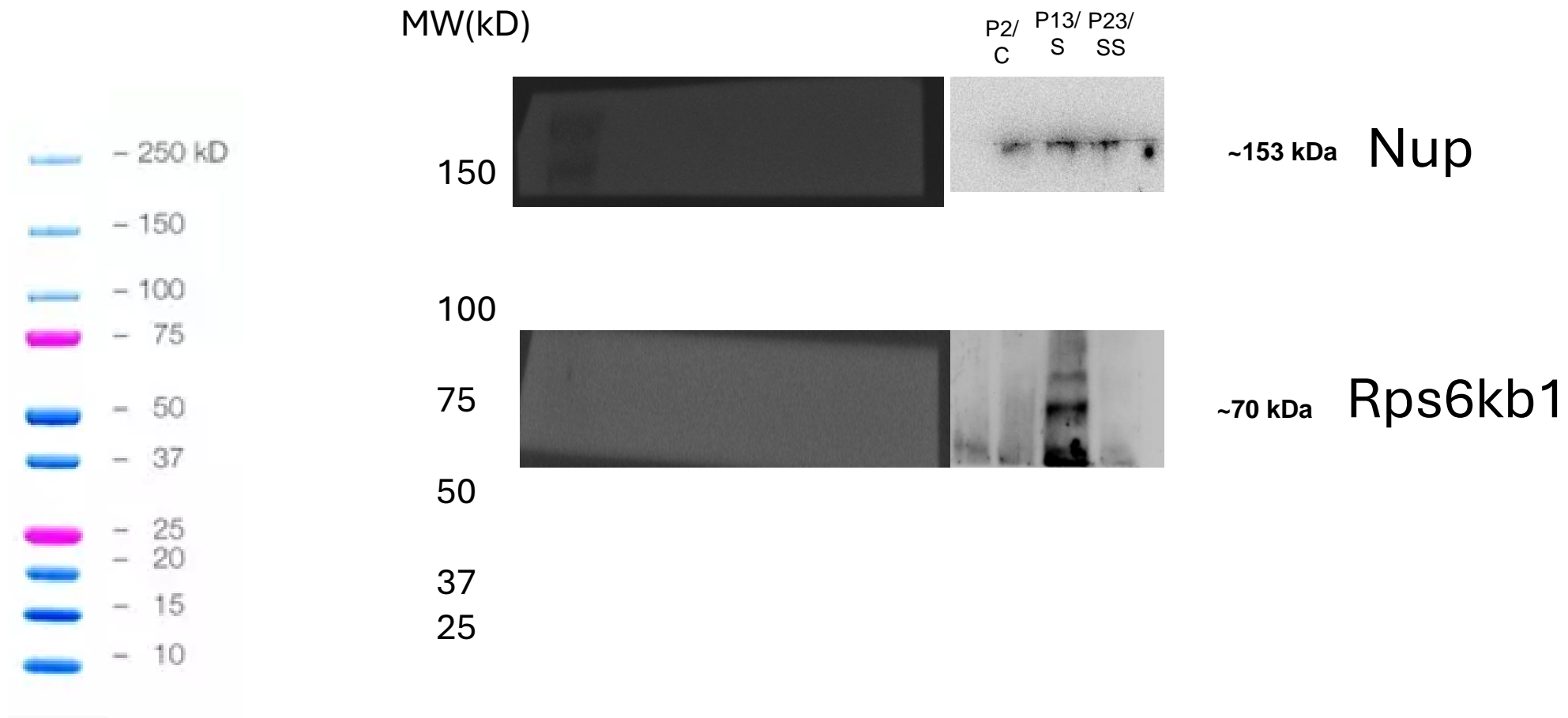

Supplement: Supplementary file 1 [file proteomes-12-00034-s001.zip › Alonso-Puyo PROTEOMES File 2 original images of wb.pdf]
